# A Machine Learning Model for the Accurate Prediction of 1-Year Survival in TAVI Patients: A Retrospective Observational Cohort Study

**DOI:** 10.3390/jcm12175481

**Published:** 2023-08-24

**Authors:** Francesco Pollari, Wolfgang Hitzl, Magnus Rottmann, Ferdinand Vogt, Miroslaw Ledwon, Christian Langhammer, Dennis Eckner, Jürgen Jessl, Thomas Bertsch, Matthias Pauschinger, Theodor Fischlein

**Affiliations:** 1 Cardiac Surgery, Cardiovascular Center, Paracelsus Medical University-Klinikum Nuremberg, 90471 Nuremberg, Germany; magnum.rottmann@stud.pmu.ac.at (M.R.); ferdinand.vogt@artemed.de (F.V.); miroslaw.ledwon@klinikum-nuernberg.de (M.L.); theodor.fischlein@klinikum-nuernberg.de (T.F.); 2 Research and Innovation Management (RIM), Team Biostatistics and Publication of Clinical Trial Studies, Paracelsus Medical University, 5020 Salzburg, Austria; wolfgang.hitzl@pmu.ac.at; 3 Department of Ophthalmology and Optometry, Paracelsus Medical University Salzburg, 5020 Salzburg, Austria; 4 Research Program Experimental Ophthalmology and Glaucoma Research, Paracelsus Medical University, 5020 Salzburg, Austria; 5 Institute of Clinical Chemistry, Laboratory Medicine and Transfusion Medicine, Paracelsus Medical University, 90471 Nuremberg, Germany; christian.langhammer@klinikum-nuernberg.de (C.L.); thomas.bertsch@klinikum-nuernberg.de (T.B.); 6 Cardiology, Cardiovascular Center, Paracelsus Medical University-Klinikum Nuremberg, 90419 Nuremberg, Germany; dennis.eckner@klinikum-nuernberg.de (D.E.); juergen.jessl@klinikum-nuernberg.de (J.J.); matthias.pauschinger@klinikum-nuernberg.de (M.P.)

**Keywords:** personalized, TAVI, machine learning, prediction, outcome, survival

## Abstract

Background: predicting the 1-year survival of patients undergoing transcatheter aortic valve implantation (TAVI) is indispensable for managing safe early discharge strategies and resource optimization. Methods: Routinely acquired data (134 variables) were used from 629 patients, who underwent transfemoral TAVI from 2012 up to 2018. Support vector machines, neuronal networks, random forests, nearest neighbour and Bayes models were used with new, previously unseen patients to predict 1-year mortality in TAVI patients. A genetic variable selection algorithm identified a set of predictor variables with high predictive power. Results: Univariate analyses revealed 19 variables (clinical, laboratory, echocardiographic, computed tomographic and ECG) that significantly influence 1-year survival. Before applying the reject option, the model performances in terms of negative predictive value (NPV) and positive predictive value (PPV) were similar between all models. After applying the reject option, the random forest model identified a subcohort showing a negative predictive value of 96% (positive predictive value = 92%, accuracy = 96%). Conclusions: Our model can predict the 1-year survival with very high negative and sufficiently high positive predictive value, with very high accuracy. The “reject option” allows a high performance and harmonic integration of machine learning in the clinical decision process.

## 1. Introduction

The introduction of transcatheter aortic valve implantation (TAVI) in the past decade has revolutionized the approach to managing patients affected by severe aortic stenosis. As compared with surgical aortic valve replacement (SAVR), crucial issues for the success of TAVI include its minimal invasiveness, avoidance of cardiopulmonary bypass and a reduced incidence of early complications, such as bleeding, especially in elderly and intermediate- to high-risk patients [1]. Moreover, the possibility of avoiding general anesthesia in most cases has raised the question of the feasibility and safety of early discharge strategies [2,3,4]. Early discharge strategies can prevent hospital-related infections as well as save both costs and resources, topics that acquired increased weight during the coronavirus-2019 (COVID-19) pandemic. On the other hand, about 10% of patients who undergo TAVI experience readmission for any cause in the first 30 days post procedure in high-volume centres [2,3]. Moreover, although the rate of 30-day mortality remains low (about 1%), 1-year mortality occurs in about 7% of patients [4]. Because there is a lack of consensus about both the timing (i.e., same or next day) and selection criteria for early discharge, tools for the personalized prediction of outcomes (as opposed to general or standardized algorithms) could be useful for improving patient eligibility. The complexity and variety of clinical scenarios in this particular population can result in a difficult prognosis for clinicians. Prior studies have highlighted the influence of clinical, anatomical and procedural variables on postprocedural complications and survival rates [5,6,7]. Nevertheless, postoperative complications and residual paravalvular regurgitation have also demonstrated a significant influence on the outcomes. Artificial intelligence and machine learning (ML) are expected to provide increasingly complex prediction models. Therefore, we aimed to develop an ML model that is able to assess the 1-year survival of patients following TAVI with a high prediction performance.

## 2. Methods

Based on our prior experience and results in identifying risk factors for outcomes after TAVI [5,6,7], the Machine Learning for Aortic Valve Interventions (MALAVI) initiative began in March 2019. The project started from the observation of the complexity of a personalized heart team evaluation of patients affected by aortic valve stenosis, as suggested by international guidelines. The general scope of the MALAVI project was to support clinicians’ decision making through the use of ML to achieve the lowest possible rate of complications. This is a monocentric, retrospective observational cohort study conducted in accordance with the STROBE statement [8], as reported in Supplementary Table S1.

For the specific scope of the present manuscript, we retrospectively analyzed our centre’s experience with TAVI procedures between January 2012 and August 2018. All consecutive patients who underwent TAVI for symptomatic severe stenosis of the native aortic valve were included in the study. The indication for TAVI was in accordance with international guidelines, that is, in patients at intermediate and/or high or prohibitive surgical risk after discussion by the institutional heart team, which consisted of an interventional cardiologist, an imaging specialist cardiologist, a cardiac surgeon and an anesthetist [9]. Exclusion criteria were bicuspid aortic valve of type Sievers 0, pure aortic regurgitation, absence of a baseline electrocardiogram-triggered multislice computed tomography (MDCT) and use of experimental prostheses. Overall, 881 patients were evaluable. Because of the deep differences in outcomes, only patients who underwent transfemoral (TF)-TAVI were included, excluding the transapical (n = 252) and transaortic (n = 1) access. Clinical, operative and postprocedural data were prospectively collected in our institutional database. A total of 134 input variables were considered for the model. All variables and definitions are listed in Table S2. Basically, preoperative characteristics were defined based on the EuroSCORE II criteria, whereas procedural and postprocedural variables were based on the VARC-2 criteria [10,11]. The VARC-2 definition considers the onset of most complications within the first 72 h following the index procedure. The follow-up was conducted through visits in the outpatient ward and, alternatively, through telephone contact with patients or their physicians, and it was concluded in March 2022. All patients provided informed consent for the anonymous use of their data, and the study was approved by the institutional review board (IRB-2022-10). The study protocol conforms to the ethical guidelines of the Declaration of Helsinki.

To improve negative and positive predictive powers of the models, an important and common method in machine learning—the “reject option”—was applied. This means that the models were allowed to refuse to make a prediction (i.e., the model was “in doubt”). The advantage of this option is that it substantially increases the accuracy of the models in such a way that the medical expert can safely rely on the prediction. The disadvantage of this method, however, is that 19% of individuals do not receive a prediction. If no prediction is made with the model, a prediction concerning the 1-year survival can still be made by the clinical expert if desired.

Medical expert knowledge was used together with the results of published studies to select a large set of possible candidate predictors expected to have high or moderate prediction power. These are shown in Table S2 (bold marked label).

### Statistical Methods

Data were screened and cleaned for incomplete, incorrect and missing data. Data were analyzed and tested for normality. To assess the univariate predictive power, continuously distributed data were analyzed for various distributions, including normal, log-normal and gamma distributions. Fisher’s exact test and Pearson’s chi-square test were used to analyze cross-tabulation tables. In addition, generalized linear models with log-normal and gamma distributions as well as *t*-tests with and without the assumptions of variance homogeneity were used for continuous variables.

ML Algorithms: Multilayer perceptron neural networks, support vector machines, nearest neighbour classifiers, random forest models and Bayes classifiers were built, trained in learning samples using 10-fold cross-validation and additionally tested in an independent test sample. Model performances were compared against each other using negative predictive value (NPV), positive predictive value (PPV) and accuracies. Supplementary Table S3 in the appendix presents an overview of preprocessing; model training; the split of data into training, validation and test samples; training stop criteria; selection of thresholds for the reject option and the final test of the model. No data imputation was performed to replace the missing data; all models were trained with complete data.

Feature Selection: We performed feature selection in two steps. In the first step, prior medical expert knowledge was used together with the results of published studies to reduce the number of the initial 134 input variables to a smaller set of 40 candidate predictors, which were expected to have high or moderate prediction power. Within this large set of candidate predictors, it is still highly likely that there are correlated and redundant variables in this set. In the second step, these 40 candidate predictors were then offered a genetic algorithm for variable selection [12] to further reduce the number of predictor variables. The genetic algorithms for the feature selection algorithm were directly integrated as part of the learning algorithm (the “embedded method”), which means that the genetic algorithm was applied during training.

Reject Option: To allow the algorithm to refuse a prediction (“if the prediction model is in doubt”), we applied the “reject option” [13]. This means that, in the finally trained model, two cutoffs instead of one for posterior probabilities were chosen in the training sample and then tested in the test samples. We aimed to identify prediction models with high NPVs and PPVs for two reasons: a false-negative prediction will determine the discharge of a patient who is at risk of dying within 1 year, and a false-positive prediction may lead to maintaining the unnecessary hospitalization of a patient who is not expected to die. This unnecessarily increases resource consumption and the risk of hospital-related infections. To achieve the goal of high NPVs and PPVs, all models were checked as to whether the application of the so-called “reject option” increased predictive values as compared with models without a reject option. To explain why a certain number of patients did not receive a prediction, how closely both data clouds are stuck together and thus the need for a reject option, we illustrate the overlap between various data distributions using matrix plots of univariate predictors.

A priori probabilities of the 1-year survival were used by feeding the model with the a priori distribution to increase model performance.

Model Performance: Finally, to assess model performance after applying the reject option, we computed NPVs and PPVs, the percentage of subjects without a prediction and the total of correctly predicted numbers (i.e., the number of patients with a correct prediction proportional to the number of patients receiving a prediction) in the training and test samples. To demonstrate how the algorithms generalized to the new previously unseen data, we compared and reported the results from the training and test samples [13]. All reported tests were two-sided, and *p* values < 0.05 were considered statistically significant. All statistical analyses in this report were performed using STATISTICA 13 (StatSoft, Tulsa, OK, USA) and MATHEMATICA 13 (Wolfram Research, Inc., Champaign, IL, USA) [14].

## 3. Results

A total of 629 patients undergoing TF-TAVI between January 2012 and August 2018 were included in the study. Table 1 provides an overview of the demographic data. The prosthesis model and label sizes are shown in Supplementary Table S4. From 64 patients, the 1-year survival was missing, such that data from 565 patients’ data records were included for modelling. Among all TAVI patients, 88.1% (498/565) were still alive after 1 year, but 11.8% (67/565) died within 1 year. This information was used in the a priori distribution.

Univariate significant predictors: Of the 40 candidate variables, 19 were determined to be significant predictors—baseline transvalvular Dmax, *p =* 0.049; C-reactive protein (mg/dL), *p* = 0.0003; QRS complex duration (ms), *p* = 0.008; QTc interval (ms), *p* = 0.005; baseline hemoglobin level (g/dL), *p* = 0.016; baseline white blood cell count (×1000/uL), *p* = 0.033; age, *p* = 0.0006; baseline left ventricular ejection fraction, *p* = 0.0015; computed tomography (CT)-based aortic annulus area (mm^2^), *p* = 0.008; CT-based aortic annulus perimeter (mm), *p* = 0.019; SAPS2 value on intensive care unit admission, *p* = 0.0006; discharge transvalvular Dmean, *p* = 0.039; creatinine (mg/dL) postprocedural peak, *p* = 0.000006; extracardiac arteriopathy, *p =* 0.016; non-insulin-dependent diabetes mellitus, *p* = 0.02; baseline mitral valve regurgitation, *p* < 0.0001; valve prosthesis’s label size, *p* = 0.04; prior valvuloplasty, *p* = 0.02 and postprocedural paravalvular regurgitation, *p* = 0.016. An overview of the candidate predictor variables is given in Table 2.

Medical expert knowledge was used together with the results of published studies to select the set of 40 possible candidate predictors that were expected to have high or moderate prediction power. A genetic feature selection algorithm was used to further reduce the set of predictors.

Performance of Other ML Algorithms: Before applying the reject option, the model performances in terms of NPV/PPV were similar between all models. Support vector machines and random forests showed better performance (Table 3). Therefore, these models were also analyzed using the reject option to further increase NPV and PPV at the cost of accepting the situation in which some patients will not get a prediction.

**Figure 1:**
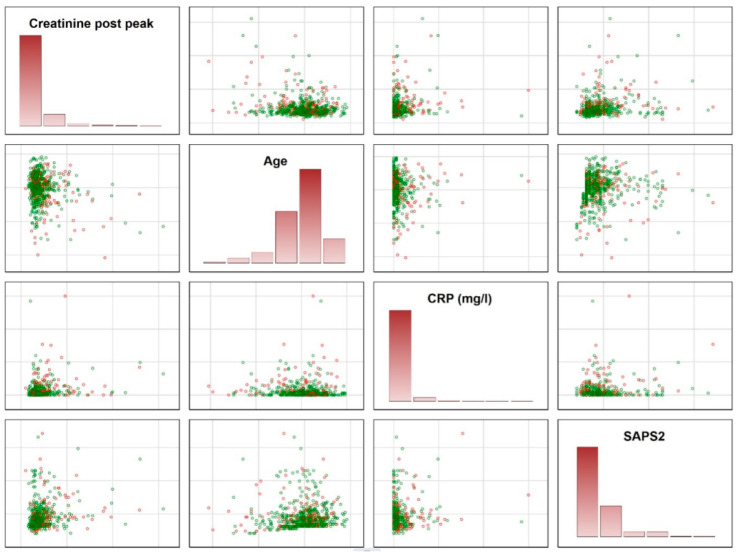
Illustration of the overlap between patients surviving and dying within 1 year. The figures demonstrate the need for a reject option, which was used to identify patients in the areas of overlap. Green circles represent the patients being alive after 1 year; the red circles show the patients dying within 1 year. It is impossible to make accurate predictions for patients in the overlap. These patients were identified using the final model and did not receive a prediction. CRP = C-reactive protein; SAPS2 = Simplified Acute Physiology Score (SAPS) II.

To explain why a certain number of patients received no prediction, we provide a deeper insight into how closely both data clouds are stuck together and illustrate the overlap between various data distributions. As illustrated, there are considerably large overlaps between various distributions, indicating the need for the reject option (Figure 1).

**Figure 2:**
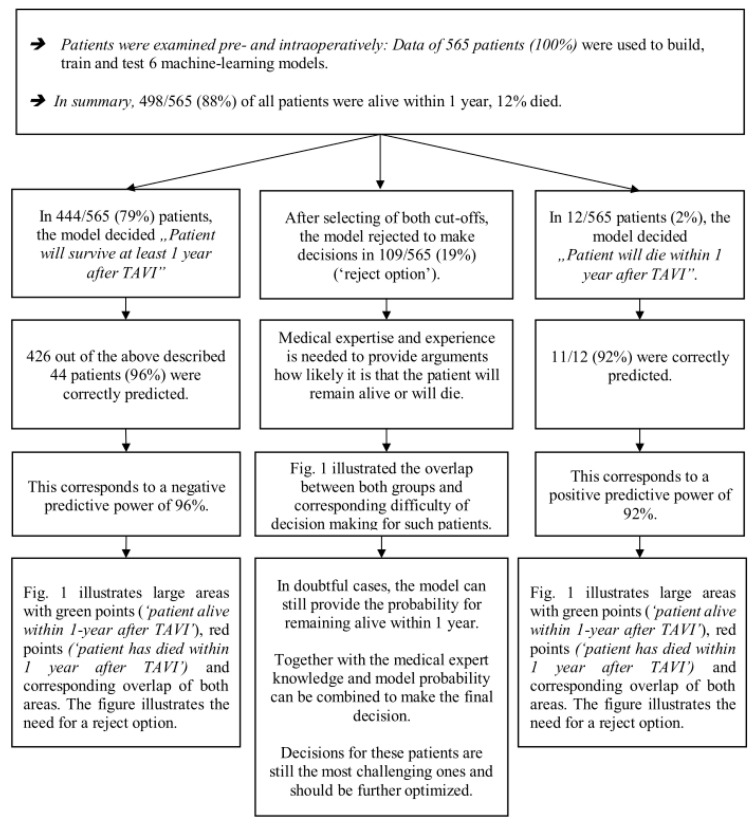
Illustration of model performance of the prediction model of 1-year survival of patients undergoing TAVI.

Model Performances Using the Random Forest Model after Applying the Reject Option: The best ML model achieved an NPV of 96% and a PPV of 92%. The algorithm excluded 109 of 565 (19%) patients, and the total correctly predicted number was also 96% (Figure 2; Table 4 and Table S5).

In the remaining 437 cases (81%) in which a prediction was made, the corresponding NPV was 96%, and the PPV was 92% (Table 4). To provide a better understanding of the models and the reject option, we provide real data (Table 5).

## 4. Discussion

Nowadays, TAVI represents the main solution—together with SAVR—for the treatment of severe aortic valve stenosis. The classical and most applied scores for the prediction of perioperative mortality—namely, EuroSCORE II and STS-PROM—were designed for SAVR, but many randomized trials conducted over the past decade have proved their inadequacy in predicting the outcome when applied to TAVI in the all-risk category [1]. If the original scope of these scores was to support the heart team in deciding between TAVI and SAVR, recent evidence showing good early and mid-term results—and in some cases also superiority—in favour of TAVI has definitely made the use of these scores obsolete. Indeed, recent guidelines focus on life expectancy and anatomical variables as the main issues for the heart team’s discussion [15]. In addition to the important issue of choosing between TAVI and SAVR, the spread of the application of TAVI uncovers new challenges for clinicians. Considering the higher prevalence of aortic stenosis in the elderly population, the application of TAVI over SAVR is reasonably expected to continue to increase. The higher costs of the transcatheter valve and concerns about the economical sustainability of TAVI as first-line treatment will be a challenge for the next decade [16]. The shorter length of stay and lower resource consumption have the potential to significantly lower hospital costs [17]. On the other hand, these strategies could significantly affect patient safety. Prior experiences with standard algorithms did not result in a statistically significant reduction in 1-year mortality (e.g., Spence and colleagues noted a reduction from 10.5% to 6.4% using 11-point Discharge Risk Evaluation criteria) [18]. Instruments used in a personalized decision process, possibly with a prediction period longer than 30 days, are essential and necessary.

The role of ML models in medicine in recognizing a complex statistical pattern is an issue of debate. To the best of our knowledge, only a few studies have investigated the performance of ML in predicting the clinical outcomes following TAVI [19,20]; however, the prediction was limited to only in-hospital outcomes. Hernandez-Suarez and colleagues, based on a large interhospital data set (namely, the National Inpatient Sample [NIS]) including 10,883 TAVI procedures, tested the performance of four different ML models in predicting all-cause in-hospital mortality: logistic regression, neural network, I Bayes and random forest [19]. The authors found that the logistic regression-based artificial intelligence had the best area under the curve (AUC = 0.92; 95% confidence interval: 0.89 to 0.95). Overall, acute kidney injury was the variable with the greatest importance across all ML algorithms, followed by cardiogenic shock, fluid and electrolyte disorders, cardiac arrest, sepsis, dyslipidemia, hypertension, coagulopathy, current smoking and vascular complications, respectively. Unfortunately, the variable’s definition was based on the ICD-9 cm and not on the VARC criteria, thus generating possible bias, especially regarding the clinical variable. In fact, although this method may be highly accurate regarding procedures (for billing purposes), some nonrelated clinical diagnoses may be omitted and may not represent the true prevalence of risk factors, whereas the anatomical variables are completely not evaluated. Moreover, the time of onset of some variables (such as cardiogenic shock) is not well defined and could refer both to baseline condition and post procedure. Finally, their study population included patients up to 2015, thus excluding the intermediate-risk population.

Gomes et al. recently published their experience using three different models (i.e., neural networks, support vector machines and random forests) in predicting in-hospital complications such as all-cause mortality, stroke, major vascular complications, paravalvular leakage and new pacemaker implantations [20]. In contrast to Hernandez-Suarez and colleagues, their data set was based on a single-centre experience with 451 patients, including 83 variables (including clinical, electrocardiogram, echocardiograph, CT, laboratory variables and postprocedural complications) defined based on VARC criteria.

However, the data set of Gomes et al. showed a predominance of echocardiographic parameters, whereas some important information, such as CT-based annuli dimensions and oversizing/undersizing, was missing [20]. Those researchers found that the random forest model was the most promising for in-hospital mortality (AUC = 0.97, accuracy = 90%, sensitivity = 0.96), which is in accordance with our study, but they could not accurately predict other outcomes.

The limitations of the above-mentioned NIS score and Gomes score, mainly related to short-term prediction, make their use unsuitable for supporting an early discharge strategy. The novelty of our MALAVI-t1y (tavi-1-year) model could be summarized in three points: (1) it is the first ML model for patients with TAVI designed to predict the longest outcome (i.e., 1-year survival) reported in the literature to date, (2) it is the first ML model that takes into account defined and quantitative anatomical variables alongside other well-known variables and (3) it is the first model to introduce the reject option and thus achieve higher model performance.

The wider application of the TAVI to patients at lower risk, and thus with a longer life expectancy, shifts the focus to long-term outcomes. The successful implantation of a transcatheter valve in the aortic position does not reset the pathological findings of patients affected by degenerative aortic stenosis, and late-onset complications could be responsible for the outcome of death. Retained calcified leaflets can lead to leaflet thrombosis and stroke [21], as well as to residual paravalvular regurgitation, which is associated with reduced survival at 2 years [22]. Moreover, the postprocedural onset of a conduction disturbance such as a left bundle branch block (the incidence of which is about 15%) did not result in any additional risk in the short-term (30-day) follow-up but was shown to be associated with an increased risk of all-cause (19.3%) and cardiovascular (16.2%) mortality at 2 years [23]. Therefore, focusing on the mid-term follow-up seems to better address the needs of the modern TAVI population. Interestingly, some of the 19 variables which contribute to the prediction are also editable. Indeed, a low LVEF can efficiently be treated improving the medical therapy [24,25]. Anemia could be easily addressed as well as infections. The application of KDIGO guidelines (the “KDIGO bundle”, consisting of the following elements: avoidance of nephrotoxic agents, discontinuation of angiotensin-converting enzyme inhibitors and angiotensin II receptor blockers for the first 48 h after the procedure, avoidance of hyperglycemia for the first 72 h after the procedure, avoidance of radiocontrast agents, continued close monitoring of urinary output via indwelling urinary catheters, and most importantly, goal-directed fluid therapy, which focused on evidence-based hemodynamic endpoints) can reduce the frequency and the severity of acute kidney injury [26,27]. Recently, Flores-Emanzor and colleagues reported promising results in the use of percutaneous occluders to treat the moderate-to-severe residual paravalvular leaks [28]. The moderate-to-severe mitral insufficiency, if not regressed after the TAVI, can be effectively treated percutaneously, with good 5-year outcomes [29]. Addressing the above-mentioned factors, through a personalized pathway of care, they may theoretically lengthen the survival of these patients. The use of ML for guiding the long-term care of patients goes beyond the scope of the present study and should be investigated through future investigation ad hoc.

In our model, the dimension of the aortic annuli (as area and perimeter) as assessed with MDCT was correlated with survival. The role of this anatomical variable has already been widely investigated [5,6,7] and is now an important part of the heart team discussion [15]. However, its inclusion in prior ML models for TAVI has been limited. Our results stress the importance of this variable and the careful and shared discussion of anatomical structures within the heart team.

To the best of our knowledge, our MALAVI-tavi-1y is the first ML model for patients with TAVI that uses the reject option, which allows the model to achieve higher NPV and PPV (visual abstract). The goal to find a model with high NPV and PPV was achieved, however, at the cost that 19% of all patients did not receive a prediction. Thus, we suggest that in follow-up studies, additional predictors should be identified to build models to decrease this number, on the one hand, and to end up with high NPV and PPV, on the other. The classical version of the old scores, which expresses the risk in a percentage, always returns a value; however, it is not always simple to apply for dichotomy outcomes (such as mortality) and prolongs the discussion. Physicians cannot just discharge four-fifths of a single patient alive, while the remaining fifth of that person dies—the patient either lives or dies. The establishment of cut-off values leads to inaccuracies and failure of prediction. The purpose of the reject option is to support the clinician in the decision process. If the MALAVI-t1y is “not sure” about the prediction, it does not make a prediction; this is a sort of the mathematical equivalent of the journalistic “no comment”. This occurs in about one-third of patients, whereas in the other two-thirds, the model makes highly accurate predictions. Considering that the ML is not intended to be a substitute for clinical decision making but rather to support it, we find this to be an acceptable compromise. The clinical decision remains the domain of the clinicians within a co-operative relationship with the patient.

## 5. Strengths and Limitations of This Study

Strengths: The most important strength of this study is that it provides a prediction model for patients with TAVI with excellent accuracy to identify patients who will survive within 1 year. In addition, the models are not only theoretical concepts but can also be implemented in any database and are of practical use in the clinic to support the clinician’s decision. The large sample size of about 565 patients is also an important strength of the study because a large sample size is important for resulting in statistically sound models, as emphasized by mathematical statistics and ML theory. It is important that the models were 10-fold cross-validated in the learning sample and, after application of the reject option, also tested in an independent test sample to avoid overlearning and to provide accurate predictions when the models are confronted with new, previously unseen data (generalization to new data and model stability).

Limitations: Some limitations must be disclosed. This algorithm is based on retrospective data from a single-centre experience. Although the TAVI procedure is quite standardized worldwide, and our results are in line with those reported in the international literature, external validation and prospective observational (not interventional) studies are recommended. Our group is currently working on both. The study population predominately consisted of high- and intermediate-risk patients under general anesthesia, as at the time of the study period the TAVI procedure was not yet endorsed for low-risk or younger patients. In addition, if 1-year mortality in the latter group is expected to be lowered even further, future studies should also investigate the reliability in this specific category of patients. The same consideration is valid for procedures conducted with local anesthesia, which now represents the standard of care. To conclude, readers should be aware that all included patients were selected from a heart team in accordance with the guidelines, meaning that each patient was assigned to either TAVI or SAVR in order to achieve a higher performance and achieve the lowest complication rate possible. This selection a priori is also fundamental for the correct functioning of the MALAVI model.

## 6. Conclusions

Our machine learning model identifies a subcohort of 81% of the original cohort and predicts 1-year mortality with an accuracy of 96%. The NPV of the final model based on random forests was 96% and the PPV was 92% after applying the reject option. Decisions for the remaining 19% of patients are still challenging, and it is up to the clinical expert to make a prediction. This finding will support the heart team in selecting patients eligible for early discharge strategies. These results may improve early discharge strategies and secure resource distribution while not impairing patient safety.

## Figures and Tables

**Table 1:** Overview of the characteristics of the study population with outcomes (baseline characteristics and clinical outcome).

Characteristic	Mean or n (%)
Gender (male/female)	45%/55%
Age, y (mean, range)	81.9 (53.8–94.5)
Body mass index, kg/m^2^ (mean, range)	27.1 (16.6–45.8)
Extracardiac arteriopathy	109 (19%)
Prior cardiac surgery	82 (15%)
Recent myocardial infarction	131 (23%)
Prior PCI	164 (29%)
Dialysis, n (%)	11 (1.7%)
EuroSCORE II (mean ± SD)	7% (±7)
Coronary obstruction	5 (0.9%)
Ischemic stroke	10 (1.8%)
Life-threatening bleeding	12 (2.1%)
Major vascular complications	29 (5.1%)
Need for permanent pacemaker implantation	44 (7.8%)
Procedural mortality, n (%)	7 (1.2%)
In-hospital mortality, n (%)	24 (4.2%)
ICU stay in days (mean, range)	2.0 (0–43)
Hospital stay in days (mean, range)	10.25 (0–91)
Follow-up time in days (mean, range)	927.1 (0–2665)
1-year mortality, n (%)	67 (10.2%)

**Table 2:** Overview of candidate predictor variables used for the algorhythm, according to the outcome.

	Mean	Mean	Std	Std	
Predictor	Survived within 1 Year	Died within 1 Year	Survived within 1 Year	Died within 1 Year	*p*-Value
Continuous Variable					
Age (years)	82.2	80	5.6	8.29	0.0006
Indexed effective orifice area	0.4	0.4	0.09	0.1	0.18
Body mass index	27.2	26.7	4.77	4.76	0.45
Body surface area	1.8	1.8	0.22	0.25	0.54
Estimated creatinine clearance (mL/min)	48.1	44.7	18.71	22.11	0.24
Creatinine (mg/dL) postprocedural peak	1.26	1.76	0.80	1.06	0.000006
Serum C-reactive protein (mg/dL)	0.7	1.9	1.42	3.13	0.0003
CT-based aortic annulus perimeter (mm)	76.5	78.8	7.4	8.7	0.019
CT-based aortic annulus area (mm^2^)	4.5	4.8	0.88	1.04	0.008
DLZ calcium load (mm^3^)	879.9	903.9	630.24	672.27	0.78
Eccentricity index	0.2	0.2	0.07	0.07	0.9
Baseline transvalvular Dmax	76.8	69.8	24.06	26.34	0.049
Baseline transvalvular Dmean	45.4	42.2	14.43	17.56	0.17
Baseline LV ejection fraction	54.3	49.2	12.06	14.32	0.0015
Baseline hemoglobin (g/dL)	12.3	11.8	1.74	1.96	0.016
Baseline hematocrit (%)	37.2	36.1	4.81	5.47	0.13
Grade of oversizing (%)	0.2	0.1	0.18	0.23	0.53
Platelet count (×1000/uL)	219.3	227.9	69.66	92.25	0.47
PR interval (ms)	173.8	179.9	40.88	36.56	0.38
QRS duration (ms)	99	108	24.16	29.36	0.008
QTc interval (ms)	447.5	465.7	35.72	46.11	0.005
SAPS2	25.9	31.1	10.98	14.22	0.0006
Calcium load of aortic valve (mm^3^)	815.7	814.6	583.85	649.9	0.99
Calcium load of LVOT (mm^3^)	64.2	89.3	101.72	113.37	0.09
White blood cell count (×1000/uL)	7.1	7.9	2.29	3.08	0.033
Discharge transvalvular Dmean	11.1	9.6	4.67	3.89	0.039
Categorical Variable					
	Survived within 1 Year		Died within 1 Year		
Gender (M/F)	45%, 55%		57%, 43%		0.066
Extracardiac arteriopathy	17.8%		30.3%		0.016
Non-insulin-dependent diabetes mellitus	28.1%		41.8%		0.02
New York Heart Association class (I, II, III, IV)	0.8%, 11.2%, 74.5%, 13.6%		1.5%, 7.6%, 65.2%, 25.7%		0.053
Baseline aortic valve insufficiency †	25%, 58.7%, 15.8%, 0.5%		30.9%, 49.1%, 16.4%, 3.6%		0.057
Baseline mitral valve insufficiency †	13.1%, 64.9%, 21.1%, 0.9%		5.7%, 56.7%, 22.5%, 15.1%		<0.0001
Baseline tricuspid valve insufficiency †	45.4%, 42.3%, 9.4%, 2.9%		52.6%, 29.9%, 8.8%, 8.8%		0.061
Persistent/permanent atrial fibrillation	29.9%		41.8%		0.067
Valve prosthesis (type) §	0%, 20.1%, 4.8%, 3.8%, 3.2%, 21.5%, 46.6%		1.5%, 17.9%, 10.5%, 7.5%, 3%, 17.9%, 41.7%		0.052
Valve prosthesis’s label size ‡	0%, 34.7%, 8.9%, 34.1%, 4.8%, 12.5%, 3.4%, 1.4%		1.5%, 26.9%, 6%, 29.8%, 4.5%, 22.4%, 7.4%, 1.5%		0.04
Valvuloplasty prior to prosthesis implantation	98.4%		94.3%		0.02
Postprocedural paravalvular regurgitation †	73.6%, 21.9%, 4.5%, 0%		70.8%, 18.8%, 8.3%, 2.1%		0.016

† Classified in 4 categories, respectively: none, mild, moderate and severe. § The model’s name is listed in Supplementary Table S3. ‡ Classified in 8 categories, respectively: 0, 23, 25, 26, 27, 29, 31 and 34 mm.

**Table 3:** Overview of the model performances of five machine learning algorithms to predict 1-year survival before application of the reject option. Model comparisons are based on negative and positive predictive values. Results are based on 10-fold cross-validation.

Model Performance	Support Vector Machine	Nearest Neighbour	Neuronal Network	Bayes Classifier	Random Forest
AUC	74%	72%	78%	82%	81%
NPV/PPV	88%/— ^1^	87%/—	91%/71%	90%/81%	93%/—

Model performances are very similar between all models. Random forest models and support vector machine models showed the most promising results in terms of receiver operating characteristic curves and were further analyzed using the reject option (Table 4). NPV = negative predictive value; PPV = positive predictive value. ^1^ No estimation of PPV was possible.

**Table 4:** Overview of model performances after applying the reject option to the random forest models’ predictions of 1-year survival of patients undergoing TAVI.

	Negative Predictive Power	Positive Predictive Power	Unpredicted	Total Correctly Predicted
Training sample (10-fold cross-validation)	(305/315) 98%	(11/12) 92%	(68/395) 17%	(316/325) 97%
Test sample	(121/129) 94%	(0/0) -	(41/170) 24%	(121/129) 94%
Overall sample	(426/444) 96%	(11/12) 92%	(109/565) 19%	(437/456) 96%

**Table 5:** Illustration of model performance for predicting 1-year survival for eight patients undergoing TAVI. Green highlighted patients (columns) were correctly predicted, and red highlighted patients were falsely predicted; patients marked in grey did not receive a prediction due to the reject option. NPV was 96%, PPV was 92% and the total correctly predicted number was 96% after application of the reject option (Table 4). The cost for this improvement is that 19% of all patients (marked in grey) did not receive a prediction.

ID	1	2	3	4	5	6	7	8
Age (years)	83.2	82.1	80.1	79.0	79.0	82.8	84.6	82.2
Hemoglobin (g/dL)	14.2	12.1	11.8	14.1	12.3	12.8	12.6	12.6
White blood cell count (×1000/uL)	6.9	7.3	4.5	6.0	7.0	6.9	6	5.7
C-reactive protein (mg/dL)	0.0	0.0	0.6	0.4	0.5	2.6	0.5	0.5
Baseline left ventricular ejection fraction	60	75	30	60	60	62	55	55
QRS duration (ms)	94	94	82	84	80	88	84	76
QTc interval (ms)	460	435	412	427	420	399	458	435
CT aortic annulus area (cm^2^)	4.8	3.8	6.1	4.1	4.9	3.5	3.59	4.3
CT aortic annulus perimeter (mm)	79.2	73.4	88.7	72.3	80.3	66.8	71.2	73.4
Creatinine postprocedural peak (mg/dL)	1.1	1.1	0.6	1.6	0.8	0.9	0.99	1.0
Gender	Male	Male	Male	Male	Female	Female	Female	Female
Extracardiac arteriopathy	No	Yes	Yes	No	No	No	0	No
Non-insulin-dependent diabetes mellitus	No	No	No	No	No	No	0	No
New York Heart Association class	II	III	II	III	III	III	III	I
Baseline persistent/permanent AF	No	No	Yes	No	No	Yes	0	No
Postprocedural aortic regurgitation	No	No	Mild	Mild	None	Trace	Trace	No
Observed 1-year survival: alive (yes/no)	Yes	Yes	Yes	Yes	Yes	No	Yes	Yes
Predicted 1-year survival: alive (yes/no/no prediction)	Yes	Yes	No prediction	Yes	Yes	No prediction	No	Yes

## Data Availability

The data sets used and/or analyzed during the current study are available in anonymized form from the corresponding author upon reasonable request.

## Supplementary Materials

The following supporting information can be downloaded at https://www.mdpi.com/article/10.3390/jcm12175481/s1, Table S1. STROBE Statement. Table S2. List of all variables used for MALAVI project. Table S3. Overview of preprocessing, model training, split of data into training, validation and test sample, model performance and final test of the model. Table S4. An overview of label sizes according to implanted prosthesis. One patient died intraoperatively after balloon dilatation and before prosthesis implantation because of annulus rupture. Table S5: Overview of model performance with various combinations of cutoffs for the random forest model.

## Abbreviations

CI = confidence interval; HR = hazard ratio; LVOT = left ventricular outflow tract; MALAVI = machine learning for aortic valve intervention; MDCT or CT = multidetector computed tomography; ML = machine learning; TAVI = transcatheter aortic valve implantation; SAVR = surgical aortic valve replacement.
