# Supplementary material for: A Machine Learning Model for the Accurate Prediction of 1-Year Survival in TAVI Patients: A Retrospective Observational Cohort Study
Source: J Clin Med. 2023 Aug 24;12(17):5481. doi: 10.3390/jcm12175481 (PMC10488486; doi:10.3390/jcm12175481)
Supplement: Supplementary file 1 [file jcm-12-05481-s001.zip › jcm-2522851-supplementary.pdf]

Supplementary Materials

Table S1. STROBE Statement.

	Item No	Recommendation	Page No
**Title and abstract**	1	(*a*) Indicate the study's design with a commonly used term in the title or the abstract<br>(*b*) Provide in the abstract an informative and balanced summary of what was done and what was found	1-2
**Introduction**			
Background/rationale	2	Explain the scientific background and rationale for the investigation being reported	4
Objectives	3	State specific objectives, including any prespecified hypotheses	4
**Methods**			
Study design	4	Present key elements of study design early in the paper	5
Setting	5	Describe the setting, locations, and relevant dates, including periods of recruitment, exposure, follow-up, and data collection	5
Participants	6	(*a*) Give the eligibility criteria, and the sources and methods of selection of participants. Describe methods of follow-up<br>(*b*) For matched studies, give matching criteria and number of exposed and unexposed	5
Variables	7	Clearly define all outcomes, exposures, predictors, potential confounders, and effect modifiers. Give diagnostic criteria, if applicable	5
Data sources/ measurement	8*	For each variable of interest, give sources of data and details of methods of assessment (measurement). Describe comparability of assessment methods if there is more than one group	5
Bias	9	Describe any efforts to address potential sources of bias	5
Study size	10	Explain how the study size was arrived at	5
Quantitative variables	11	Explain how quantitative variables were handled in the analyses. If applicable, describe which groupings were chosen and why	6
Statistical methods	12	(*a*) Describe all statistical methods, including those used to control for confounding<br>(*b*) Describe any methods used to examine subgroups and interactions<br>(*c*) Explain how missing data were addressed<br>(*d*) If applicable, explain how loss to follow-up was addressed<br>(*e*) Describe any sensitivity analyses	6
**Results**			
Participants	13*	(a) Report numbers of individuals at each stage of study—eg numbers potentially eligible, examined for eligibility, confirmed eligible, included in the study, completing follow-up, and analysed<br>(b) Give reasons for non-participation at each stage<br>(c) Consider use of a flow diagram	8
Descriptive data	14*	(a) Give characteristics of study participants (eg demographic, clinical, social) and information on exposures and potential confounders<br>(b) Indicate number of participants with missing data for each variable of interest<br>(c) Summarise follow-up time (eg, average and total amount)	Table 1 and S3

Outcome data	15*	Report numbers of outcome events or summary measures over time	8

Main results	16	(*a*) Give unadjusted estimates and, if applicable, confounder-adjusted estimates and their precision (eg, 95% confidence interval). Make clear which confounders were adjusted for and why they were included<br>(*b*) Report category boundaries when continuous variables were categorized<br>(*c*) If relevant, consider translating estimates of relative risk into absolute risk for a meaningful time period	Table 1 and S3
Other analyses	17	Report other analyses done—eg analyses of subgroups and interactions, and sensitivity analyses	9
**Discussion**			
Key results	18	Summarise key results with reference to study objectives	10
Limitations	19	Discuss limitations of the study, taking into account sources of potential bias or imprecision. Discuss both direction and magnitude of any potential bias	13
Interpretation	20	Give a cautious overall interpretation of results considering objectives, limitations, multiplicity of analyses, results from similar studies, and other relevant evidence	10-12
Generalisability	21	Discuss the generalisability (external validity) of the study results	10-12
**Other information**			
Funding	22	Give the source of funding and the role of the funders for the present study and, if applicable, for the original study on which the present article is based	15

*Give information separately for exposed and unexposed groups.

Note: An Explanation and Elaboration article discusses each checklist item and gives methodological background and published examples of transparent reporting. The STROBE checklist is best used in conjunction with this article (freely available on the Web sites of PLoS Medicine at http://www.plosmedicine.org/, Annals of Internal Medicine at http://www.annals.org/, and Epidemiology at http://www.epidem.com/). Information on the STROBE Initiative is available at http://www.strobe-statement.org.

Table S2. List of all variables used for MALAVI project.

Name	*Type*	*Description*
**Age (years)**	c	
**Gender**	d	0=male, 1=woman
Height (cm)	c	
Weight (kg)	c	
**BMI**	c	
**BSA**	c	
Baseline serum creatinine (mg/dL)	c	
**Baseline creatinine clearance**	c	Estimated
**Baseline chronic dialysis**	d	0=good; 1=bad
**Extracardiac arteriopathy**	d	0=good; 1=bad
Poor mobility	d	0=good; 1=bad
**Baseline Hemoglobin (g/dL)**	c	
**Baseline Hematocrit (%)**	c	
Baseline Red blood cell count (x1000000/uL)	c	
**Baseline White blood cell count (×1000/uL)**	c	
**Baseline Platelet Count (x1000/uL)**	c	
Baseline Troponin T (ng/dL)	c	
**Baseline serum C-reactive protein (mg/dL)**	c	
**Prior cardiac surgery**	d	0=good; 1=bad
**Prior CABG**	d	0=good; 1=bad
Prior valvuloplasty	d	0=good; 1=bad
Prior mitral valve replacement	d	0=good; 1=bad
Prior mitral valve repair	d	0=good; 1=bad
**Chronic obstructive pulmonary disease**	d	0=good; 1=bad
**Baseline critical state**	d	0=good; 1=bad
**Insulin-dependent diabetes mellitus**	d	0=good; 1=bad
**Non–insulin-dependent diabetes mellitus**	d	0=good; 1=bad
**Class NYHA**	d	1-4
CCS class 4	d	0=good; 1=bad
**Recent myocardial infarction**	d	0=good; 1=bad
**Baseline left ventricular ejection fraction (%)**	c	
**Severe pulmonary hypertension**	d	Assessed at preoperative TTE
PAPS (mmHg)	c	Assessed at preoperative TTE or Cardiac catheterization
**Baseline transvalvular gradient Pmax (mmHg)**	c	Assessed at preoperative TTE
**Baseline transvalvular gradient Pmean (mmHg)**	c	Assessed at preoperative TTE
**Aortic Valve Area (cm^2) indexed**	c	Assessed at preoperative TOE
Bicuspid aortic valve	d	0=good; 1=bad
**Baseline aortic valve regurgitation**	d	0-3

**Baseline mitral valve regurgitation**	d	0-3
**Baseline tricuspidal valve regurgitation**	d	0-3
Urgency	d	1=elective, 2=urgent, 3=emergency
Number of concomitant procedures (including TAVI)	d	
**Prior Percutaneous Coronary Intervention**	d	0=good; 1=bad
Baseline sinus rhythm	d	0=bad, 1=good
Baseline paroxysmal atrial fibrillation	d	0=good; 1=bad
**Baseline pers/permanent atrial fibrillation**	d	0=good; 1=bad
Baseline paced rhythm	d	0=good; 1=bad
**Prior permanent pacemaker**	d	0=bad; 1=good
Heart rate (n/min)	c	Assessed at preoperative ECG
Sinus rhythm	d	Assessed at preoperative ECG
Atrial fibrillation	d	Assessed at preoperative ECG
Left anterior hemiblock	d	Assessed at preoperative ECG
**Left bundle branch block**	d	Assessed at preoperative ECG
**Right bundle branch block**	d	Assessed at preoperative ECG
**PR interval (ms)**	c	Assessed at preoperative ECG
**QRS duration (ms)**	c	Assessed at preoperative ECG
**QTc interval (ms)**	c	Assessed at preoperative ECG
Q waves	d	Assessed at preoperative ECG
Inverted T waves	d	Assessed at preoperative ECG
Aortic annulus diameter Max (mm)	c	Assessed at preoperative MDCT
Aortic annulus diameter Min (mm)	c	Assessed at preoperative MDCT
**Aortic annulus surface (cm^2)**	c	Assessed at preoperative MDCT
**Aortic annulus perimeter (mm)**	c	Assessed at preoperative MDCT
Distance right coronary artery-annulus (mm)	c	Assessed at preoperative MDCT
Distance left coronary artery-annulus (mm)	c	Assessed at preoperative MDCT
**Oversizing (%)**	c	Assessed at preoperative MDCT
**Eccentricity index**	c	Assessed at preoperative MDCT
**Device Landing Zone's calcium load (mm^3)**	c	Assessed at preoperative MDCT
**Total AV calcium (mm^3)**	c	Assessed at preoperative MDCT
LCC calcium AV (mm^3)	c	Assessed at preoperative MDCT
RCC calcium AV (mm^3)	c	Assessed at preoperative MDCT
NCC calcium AV (mm^3)	c	Assessed at preoperative MDCT
**Total calcium LVOT (mm^3)**	c	Assessed at preoperative MDCT
LCC calcium LVOT (mm^3)	c	Assessed at preoperative MDCT
RCC calcium LVOT (mm^3)	c	Assessed at preoperative MDCT
NCC calcium LVOT (mm^3)	c	Assessed at preoperative MDCT
Additive Euroscore	c	
Logistic Euroscore	c	
Euroscore II	c	
Access for THVI	d	1=transfemoral access; 2=transapical access
**Prosthesis's type**	d	See Table S4
**Prosthesis's label size (mm)**	d	
Valve-in-valve	d	Always 0 as per protocol's definition
**Valvuloplastique before implantation**	d	

Valvuloplastique balloon mm	c	
Valvuloplastique numbers of attemps	d	
Valve deployment under rapid pacing	d	
**Balloon dilatation after deployment**	d	0=good; 1=bad
**Conversion to surgery**	d	0=good; 1=bad
Unplanned cardiopulmonary bypass	d	0=good; 1=bad
**Coronary obstruction**	d	0=good; 1=bad
**Valve malpositioning**	d	0=good; 1=bad
Second prosthesis implantation	d	0=good; 1=bad
Unplanned intraoperative PCI	d	0=good; 1=bad
**SAPS2**	c	
Intensive care unit stay (days)	c	
Need for postprocedural intraaortic balloon pump	d	0=good; 1=bad
**Periprocedural myocardial infarction**	d	0=good; 1=bad
**Spontaneous myocardial infarction**	d	0=good; 1=bad
Stroke ischemic	d	0=good; 1=bad
Stroke hemorrhagic	d	0=good; 1=bad
**Stroke disabling**	d	0=good; 1=bad
Stroke non-disabling	d	0=good; 1=bad
**Bleeding life-threating**	d	0=good; 1=bad
**Bleeding major**	d	0=good; 1=bad
Bleeding minor	d	0=good; 1=bad
Red blood units (n)	c	
Fresh frozen plasma units (n)	c	
Platelets units (n)	c	
Pericardial effusion	d	0=good; 1=bad
Pleura effusion	d	0=good; 1=bad
Serum creatinine post Peak (mg/dL)	c	the higher, the worst
**Postprocedural Acute Kidney Injury**	d	
**Vascular major complication**	d	0=good; 1=bad
Vascular minor complication	d	0=good; 1=bad
**New onset of intraventricular conduction disturbance**	d	E.g.: (LSB / RSB)
**Postoperative atrioventricular block**	d	
Postoperative permanent pacemaker implantation	d	
New onset of postprocedural atrial fibrillation	d	
New onset of others conduction disturbance	d	
**Grad of paravalvular prosthesis regurgitation intraop**	d	0-3, as assessed intraprocedural with TOE
Postprocedural transvalvular gradient Pmax (mmHg)	c	Measured with TTE
**Postprocedural transvalvular gradient Pmean (mmHg)**	c	Measured with TTE
Grad of postprocedural prosthesis regurgitation	d	0-3, measured with TTE
Type of postprocedural prosthesis regurgitation	d	1=central, 2=paravalvular, measured with TTE
Length of hospital stay (days)	c	
**Devise success**	d	0=bad, 1=good
Early safety (at 30 days)	d	0=bad, 1=good
Immediate procedural mortality	d	0=alive; 1=death

In-hospital mortality	d	0=alive; 1=death
**30-days mortality**	**d**	**0=alive; 1=death**
Length of follow-up (years)	c	
Status at last follow-up	d	0=alive; 1=death
**1-year mortality**	**d**	**0=alive; 1=death**

c = continuous; d = dichotomous

Table S3. Overview of preprocessing, model training, split of data into training, validation and test sample, model performance and final test of the model.

	Description		*Remarks*
Input normalization and encoding	Continuously distributed variables were standardized having mean 0 and unit variance	Discrete variables were encoded using one hot encoding	Preprocessing of input data before model training
Split of data into training, validation and test samples	Full sample (N = 565) was randomly split into a training sample (n = 395) and test sample (n = 170). Tenfold cross-validation was used for model training using 10% of the training data as a validation set each. No data of the test sample were used for model training.	After model training, two thresholds for the reject options were estimated to maximize negative and positive predictive values and to minimize the number of unpredicted cases.	These thresholds were applied, and negative and positive predictive values were computed in the training samples and finally evaluated in the independent and randomly selected test sample (n = 170, Table 3).
Model performances in training data	Negative and positive predictive values, percentage of unclassified subjects and total correctly predicted values were computed based on 10-fold cross-validation in the training samples for all models (Table 2).		
Final test and comparison of results of 10-fold cross-validation with independent test sample	Trained random forest models with two thresholds allowing the reject option were independently tested in the test sample. Negative and positive predictive power and the percentage of unclassified subjects were compared in the training and test samples. The performances were similar, suggesting no overfitting (Table 3).		

Table S4. An overview of label sizes according to implanted prosthesis. One patient died intraoperatively after balloon dilatation and before prosthesis implantation because of annulus rupture.

	n	Mean	SD	Median	Minimum	Maximum
0	1	0.00		0	0	0
Acurate Neo	128	24.75	1.53	25	23	27
CoreValve	33	30.09	1.70	31	26	31
Evolute R	29	29.66	2.99	29	26	34
Evolute R Pro	20	27.50	1.54	28	26	29
SapienXT	125	25.57	2.11	26	23	29
Sapien3	293	25.07	2.11	26	23	29
Total	629	25.62	2.70	26	0	34

Table S5: Overview of model performance with various combinations of cutoffs for the random forest model.

Lower Cutoff c1	Upper Cutoff c2	Negative Predictive Value (%)	Positive Predictive Value (%)	Percentage of Unpredicted Cases (PUC) (%)	Remarks
0.5	0.5	88%	—	0%	This corresponds to the model without using a reject option.
0.3	0.8	88%	—	0%	NPV and PPV did not change much, indicating that the second cutoff c2 was too large. The PUC did not increase, and thus upper cutoff c2 was considerably reduced.
0.1	0.3	90%	—	2%	NPV remained stable, PPV was slightly increased. PUC slightly increased.
0.05	0.2	93%	100%	7%	Both NPV and PUC increased.
0.02	0.1	97%	100%	29%	NPV, PPV and PUC significantly increased.
0.025	0.09	96%	92%	19%	Final decision about the cutoffs and correspondingly percentage of unpredicted cases was done after intensive discussions with the medical experts about their suggestions concerning NPV and PPV.

Before the application of the reject option, NPV was 88% and PPV could not be computed, which corresponds to the initial model. Table 2, c1 = c2 = 0.5. NPV and PPV increased to 96% and 91%, respectively, after applying the reject option and final decision of both cutoffs (corresponding to the model with the reject option, Table 3). The cost for this improvement is that 19% of all patients did not receive a prediction. Both selected cutoffs c1 and c2 were applied in a training sample and tested in the test sample to check whether the results remained stable (Table 3).
